# Nuclear Receptors as Regulators of Pituitary Corticotroph Pro-Opiomelanocortin Transcription

**DOI:** 10.3390/cells9040900

**Published:** 2020-04-07

**Authors:** Dongyun Zhang, Anthony P. Heaney

**Affiliations:** 1 Department of Medicine, David Geffen School of Medicine, University of California, Los Angeles, CA 90095, USA; dongyunzhang@mednet.ucla.edu; 2 Department of Neurosurgery, David Geffen School of Medicine, University of California, Los Angeles, CA 90095, USA

**Keywords:** adrenocorticotropic hormone, corticotroph, glucocorticoids, hypothalamic–pituitary–adrenal axis, nuclear receptor, pro-opiomelanocortin

## Abstract

The hypothalamic–pituitary–adrenal (HPA) axis plays a critical role in adaptive stress responses and maintaining organism homeostasis. The pituitary corticotroph is the central player in the HPA axis and is regulated by a plethora of hormonal and stress related factors that synergistically interact to activate and temper pro-opiomelanocortin (POMC) transcription, to either increase or decrease adrenocorticotropic hormone (ACTH) production and secretion as needed. Nuclear receptors are a family of highly conserved transcription factors that can also be induced by various physiologic signals, and they mediate their responses via multiple targets to regulate metabolism and homeostasis. In this review, we summarize the modulatory roles of nuclear receptors on pituitary corticotroph cell POMC transcription, describe the unique and complex role these factors play in hypothalamic–pituitary–adrenal axis (HPA) regulation and discuss potential therapeutic targets in disease states.

## 1. Introduction

**Figure 1:**
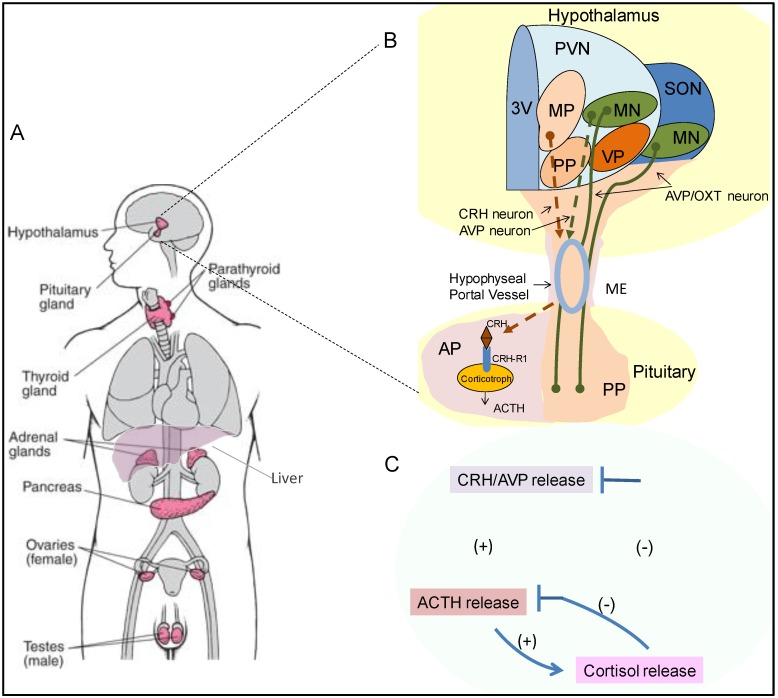
Schematic diagram of the hypothalamic-pituitary-adrenal (HPA) axis. (**A**) The major glands of the endocrine system include the hypothalamus and pituitary gland centrally and the thyroid, islet cells of the pancreas, adrenal glands, liver and gonads peripherally. (**B**) The HPA axis is regulated by hypothalamic corticotropin-releasing hormone (CRH) and arginine vasopressin (AVP), which are produced in the paraventricular nucleus (PVN) of the hypothalamus and transported to the anterior pituitary, where they drive synthesize and secretion of pituitary adrenocorticotropic hormone (ACTH). In the adrenal cortex, ACTH stimulates the adrenal zona fasciculata cortex to release glucocorticoids (GCs), which have numerous physiological effects. (**C**) GCs also exert negative feedback at the level of the hypothalamus and pituitary to dampen excessive activation of the HPA axis. 3V, the third ventricle; AP, anterior pituitary; ME, median eminence; MP, medial parvocellular; MN, magnocellular nucleus; OXT, oxytocin; PP, periventricular parvocellular; SON, supraoptic nucleus; VP, ventral parvocellular.

The pituitary gland serves as a critical anatomical and functional connection between central and multiple peripheral endocrine organs (Figure 1A). It comprises two embryonically distinct components, namely the anterior pituitary (AP) that originates from the non-neural oral ectoderm and makes up ~2/3 of the gland, and the posterior pituitary (PP) which develops from the anterior neural plate [1]. Communicating between the hypothalamus, and the adrenal, thyroid, liver, gonads and the anterior pituitary interprets fluctuating levels of neuropeptides and steroid hormones from the hypophyseal portal veins and the circulating bloodstream to coordinate production and release of six bioactive pituitary-derived peptide hormones, namely adrenocorticotropic hormone (ACTH), thyroid-stimulating hormone (TSH), follicle-stimulating hormone (FSH), luteinizing hormone (LH), growth hormone (GH), and prolactin (PRL). In contrast, the posterior pituitary is a direct extension of the hypothalamic magnocellular neurons (MN) of the paraventricular and supraoptic nuclei (PVN and SON) which travel downward along the pituitary stalk (Figure 1B). It stores and releases arginine vasopressin hormone (AVP) and oxytocin (OXT) which maintain water balance and regulate parturition and breast milk release respectively.

Arguably, the most critical component of this three-tier system is the hypothalamic–pituitary–adrenal (HPA) axis which plays a vital role in adaptive stress responses and maintaining organism homeostasis. The hypothalamic neurons in the medial parvocellular (MP) subdivision of the PVN synthesize and secrete corticotropin-releasing hormone (CRH), which travels through the hypophyseal portal vessels to reach the anterior pituitary gland (Figure 1B). CRH then binds to corticotropin releasing hormone receptor-1 (CRH-R1) which is specifically expressed on anterior pituitary corticotroph cells to stimulate the release of ACTH into the systemic circulation. The principal target for circulating ACTH is the adrenal cortex, where on binding its cognate receptor melanocortin 2 receptor (MC2R), ACTH primarily induces glucocorticoid (GC) synthesis and secretion from the zona fasciculata of the adrenal gland [2]. GCs are pleiotropic hormones, involved in almost every cellular, molecular and physiologic aspect of the organism and regulate a broad spectrum of essential physiologic functions. GCs also fine-tune HPA axis activity by inhibiting hypothalamic CRH and anterior pituitary ACTH production and secretion in a negative feedback loop manner (Figure 1C).

## 2. Pituitary Specific Regulators of Pro-Opiomelanocortin Transcription

**Figure 2:**
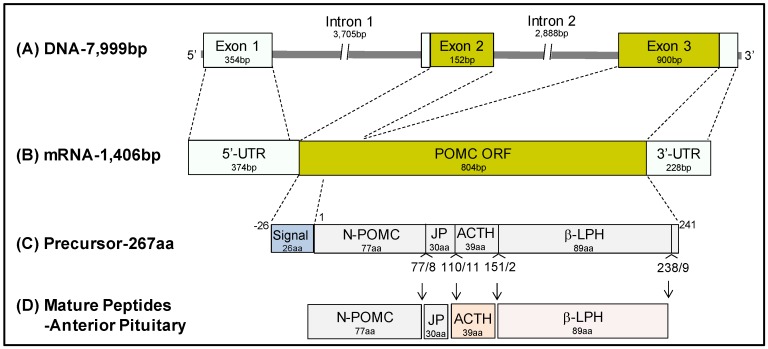
**Gene structure and the bioactive peptides derived from the human proopiomelanocortin (POMC) gene.** (**A**) The human POMC gene located on chromosome 2p23.3, spans 7999 bp on the reverse strand, and contains three exons and two introns. (**B**) The POMC transcript is processed into a 1406 bp mature mRNA with a 804 bp open reading frame (ORF). (**C**) The precursor protein of POMC contains 267 amino acids. The N-terminus 26aa signal peptide is cleaved during maturation, and the remaining region is further processed into several peptides in a tissue-specific manner. (**D**) In the anterior pituitary, POMC gives rise to a 16kDa N-POMC (also known as Pro-γ-MSH), joining peptide (JP), ACTH and beta-lipotropin (β-LPH).

Pro-opiomelanocortin (POMC) belongs to an ancestral opioid/orphanin gene family, and arose over 500 million years ago by insertion of the melanocortin sequence into a preproendorphin gene [3,4,5]. The human POMC gene is located on chromosome 2p23.3, spanning 7999 bp on the reverse strand. It contains three exons (354 bp, 152 bp, and 900 bp respectively) separated by two introns (3705 bp and 2888 bp respectively, Figure 2A). The 5′-untranslated region (5′-UTR) of POMC mRNA includes the entire Exon 1 and the first 20 bp of the proximal region of Exon 2 (Figure 2B). The remaining sequence of Exon 2 encodes a signal peptide and the first 18aa of the POMC precursor (Figure 2B). A large part of Exon 3 is translated to 223aa of the POMC peptide and the 3′-UTR covers a 228 bp sequence of the 3′ end of Exon 3 (Figure 2B). The N-terminal 26aa-signal peptide of the precursor POMC protein is cleaved during maturation, and the remaining 241aa region is further processed into several peptides in a tissue-specific manner (Figure 2C). In the anterior pituitary, POMC gives rise to several bioactive peptides, which include a 16kDa N-terminal POMC fragment (also known as Pro-γ-MSH), a joining peptide (JP), ACTH and beta-lipotropin (β-LPH) [6] (Figure 2D). Very low levels of POMC peptide and mRNA have also been identified in several extrapituitary sites, including the pancreas, stomach, appendix, kidney, testis, prostate, placenta, skin, adipose tissue, skeletal muscle, lymph nodes, brain, adrenal and lung. However the POMC transcript in these tissues differs significantly from that in the anterior pituitary in that it is shorter, contains approximately 800 bp derived from Exon 3 but lacks the N-terminus signal peptide for secretion [7]. This finding indicates that tissue specific factors are not only involved in POMC transcriptional regulation and also peptide processing. 

**Figure 3:**
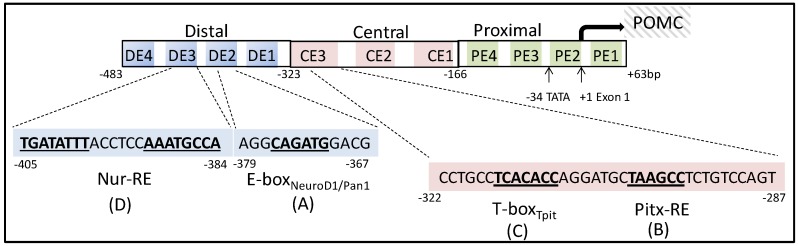
**Depiction of transcriptional regulation of POMC promoter by corticotroph-lineage specific transcription factors.** (**A**) E-box, NeuroD response element; (**B**) Pitx-RE, Pitx1 response element; (**C**) T-box, Tpit response element; (**D**) Nur-RE, Nur-factor binding element.

Alignment of the human POMC 5′-flanking promoter sequence with its rat and mouse counterparts revealed several highly conserved regions [8], which are also responsible for pituitary-specific POMC transcription [9,10]. By segmenting the rat POMC gene promoter (-483 to -34 base pairs) into distal, central and proximal regions, several DNA sequence elements that conferred pituitary specific POMC expression were identified [11]. Using mutagenesis and in vitro protein-DNA binding studies, an E-box motif present in element DE-2 of the distal domain (-379 AGG CAG ATG GAC G -367) which represented binding sites for the class I basic helix-loop-helix (bHLH) transcription factor Pan1 (also known as E47), and the class II cell-restricted bHLH factor NeuroD1 (also known as β2) was defined [12,13] (Figure 3A). Interestingly, the actions of the NeuroD1/Pan1 heterodimers on POMC transcription was augmented by an adjacent CE-3 element in the central region (-322 CCT GCC TCA CAC CAG GAT GCT AAG CCT CTG TCC AGT -287) [14]. Using this sequence as a probe to clone DNA-binding proteins from AtT20 cell cDNA expression library, the bicoid-related homeodomain protein, pituitary homeobox 1 (Pitx1), was cloned and characterized as corticotroph specific POMC transcriptional regulator [14] (Figure 3B). These authors further identified a novel T box factor, named Tpit, which binds not only the T-box efficiently, but also the Pitx1 protein physically (Figure 3C). Given the proximity of these three binding sites (E-box, T-box and Pitx-RE), it appears that the interactions between NeuorD1/Pan1, Pitx1 and Tpit form a scaffold that interacts with many other transcription factors to developmentally define and regulate cell-restricted POMC expression [15]. 

## 3. Nuclear Receptors as Pro-Opiomelanocortin Transcription Regulators in Pituitary Corticotropes

**Figure 4:**
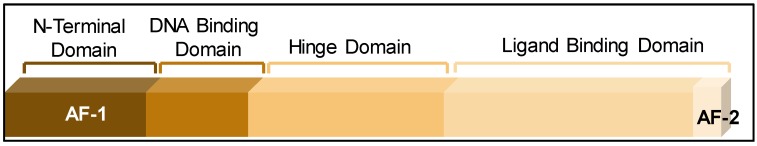
**Schematic structure of a typical nuclear receptor (NR).** The modular structure of NRs includes an N-terminal region, responsible for the ligand-independent activation function (AF-1), a DNA-binding domain, a hinge domain and a ligand-binding pocket responsible for the ligand-dependent activation function (AF-2).

Nuclear receptors (NRs) are a large group of transcription factors that are involved in many fundamental biological processes, including development, differentiation, and homeostasis [16]. From the evolutionary point-of-review, NRs emerged during the earliest metazoan period [17]. Analysis of the human genome sequence has identified 48 NR members, and sequence alignment and phylogenetic tree construction has allowed classification into six distinct evolutionary groups [18]. Currently, ~35 of these possess known ligands or activators (summarized in Table 1) [19]. NRs share 4 common structural domains, namely an N-terminal transactivation domain (NTD), a DNA binding domain (DBD), a hinge domain and a ligand binding domain (LBD) (Figure 4). Their NTD usually contains a less conserved activator function (AF-1) region, which is responsible for interaction with cell-specific transcriptional co-regulators. AF-1 activity is often regulated by post-translational modification, such as phosphorylation [20]. The DBD is the most conserved domain and usually comprises two C4-type zinc finger modules which bind to specific DNA sequences, called hormone response elements (HREs) (except for subfamily 0 members DAX1 and SHP which do not have a DBD). A classic proximal box (P-box) in the first zinc finger is responsible for DNA base recognition, and the second zinc finger in the distal box (D-box) is important for receptor dimerization followed by binding to the second half of the DNA consensus [21]. The hinge domain is the most variable region of NRs, and this flexible structure accommodates the rotation of the NR DBD to form dimers for HRE consensus binding, and provides a docking site for various co-factors. The NR LBD is a multifunctional domain that mediates not only ligand binding, but also NR dimerization in addition to its interaction with co-activator/co-repressor proteins, nuclear localization, and transactivation functions. Upon ligand binding, the AF-2 motif folds back against the core LBD, forming a novel surface for co-activator/co-repressor binding to regulate target gene transactivation. 

### 3.1. Subfamily 1

This largest NR subfamily contains 20 members belonging to 7 groups. They include the thyroid hormone receptors (NR1A1, NR1A2), retinoic acid receptors (NR1B1, NR1B2, NR1B3), peroxisome proliferator activated receptors (NR1C1, NR1C2, NR1C3), reverse Erb receptors (NR1D1, NR1D2), retinoic acid receptor-related orphan receptors (NR1F1, NR1F2, NR1F3), liver X receptors (NR1H2, NR1H3) and farnesoid X receptors (NR1H4, and NR1H5P), vitamin D receptor (NR1I1), pregnane X receptor (NR1I2) and constitutive androstane receptor (NR1I3). These receptors have well-documented ligands that control lipid and glucose homeostasis, and many have been exploited therapeutically to manage several metabolic diseases. 

#### 3.1.1. Thyroid Hormone Receptors (TRα and TRβ)

Thyroid hormones, 3,3′,5-triiodothyronine (T3) and its less active precursor 3,5,3′,5′-tetraiodothyronine (thyroxine or T4), are major regulators of development, growth, energy expenditure and reproduction. They were first recognized during study of cretinism and myxedema which resulted from loss of thyroid function [22]. Thyroid hormone deficiency causes widespread neurological and neuropsychological abnormalities during intrauterine growth and the early postnatal period. The most affected brain structures were central auditory processing and connectivity nucleus, resulting in deafness [23,24]. Thyroid hormone receptors (TRs) mediate the functions of T3/T4, and are encoded by two genes, namely TRα and TRβ, located on human chromosomes 17 and 3, respectively [25]. 

Alternative splicing of the primary transcripts gives rise to four major TR isoforms: TRα1, α2, β1, and β2 [26]. TRα1 and α2 are produced from differential usage of a splicing site inside Exon 9, generating a 40aa C-terminus (CT) domain in TRα1 and a 122aa CT domain in TRα2 [27]. Structural alteration of TRα2 CT abrogated its binding to T3/T4, resulting in weak transactivation of a subset of thyroid hormone responsive elements (TREs). TRα2 is only found in mammals, and widely co-expressed with TRα1 in embryonic and adult tissues with ~10-fold higher TRα2 than TRα1 mRNA expression [28]. TRβ1 and TRβ2 differ in their N-terminus (NT) due to alternative transcription initiation sites. The TRβ1 NT has 94aa, encoded from Exon1 and 2; while TRβ2 uses a promoter region between Exon 4 and 5 to generate a larger 147aa NT [29,30]. TRβ1 mRNA is ubiquitously expressed, whereas TRβ2 mRNA is predominantly expressed in the pituitary and hypothalamus [31,32], indicating the important role of TRβ2 in the central regulation of the hypothalamic–pituitary–thyroid (HPT) axis [33]. 

Using in situ hybridization with biotinylated complementary oligonucleotide probes to TRβ2 mRNA in dispersed rat pituitary cells, the majority of cells that expressed TRβ2 mRNA was either TSH- (56% ± 3%) or GH- (43% ± 4%) secreting cells, whereas <1% of all pituitary cells expressed TRβ2 and ACTH (0.9% ± 0.06%), LH (0.8% ± 0.1%), FSH (0.8% ± 0.1%), and/or PRL (0.9% ± 0.04%) [34]. Although there appears to be no reported direct interaction of TRα and/or TRβ with the POMC promoter, POMC^−/−^ animals displayed primary hyperthyroidism with elevated plasma T3/T4 levels, and suppressed hypothalamic TRH and pituitary TSH [35], indicating a potential as yet uncharacterized role for the melanocortin system in the regulation of the HPT axis [35]. 

#### 3.1.2. Retinoic Acid Receptors (RARα, RARβ, RARγ)

Retinoic acid (RA) is a natural bioactive metabolite of vitamin A (VA/retinol) that induces pleiotropic effects on cell growth and differentiation in both health and disease. RA bioactivity is mediated primarily by the retinoic acid receptors (RARα, RARβ and RARγ) and retinoid X receptors (RXRα, RXRβ and RXRγ) which bind to polymorphic cis-acting RA response elements (RAREs) of target genes to modulate gene transcription [36]. 

Human and higher mammals cannot synthesize RA so it must be obtained from food-intake in the form of plant-derived carotenoids (pro-RA, 60%) and animal-derived retinols (40%) [37]. Carotenoids are converted to retinals and then retinols in intestinal enterocytes, where free retinol is esterified to retinyl ester (RE) by the enzyme lecithin retinol acyltransferase (LRAT). The resulting REs are packaged into chylomicrons, which are secreted from the intestine and delivered via lymphatics to the liver [38]. When needed, RE is hydrolyzed to retinol by retinyl ester hydrolase (REH), then binds to retinol-binding protein 4 (RBP4). After mobilization from the liver, the complex is bound to thyroxine carrier transthyretin (TTR) to be transported in the general circulation. On arrival at target tissues, retinol is internalized by an RBP-binding transporter, STRA6 [39]. Cellular retinol binding protein 1 (CRBP1) serves as the chaperone of retinol inside the cell where retinol dehydrogenase (RDH) and aldehyde dehydrogenase 1 (ALDH1) sequentially oxidize retinol into RA. Three stereoisomeric forms of RA, all-trans RA (ATRA), 13cisRA, and 9cisRA are non-enzymatically isomerized between ATRA and 9cisRA/13cisRA isomers [39]. The RAR family is activated by both ATRA and 9cisRA, whereas the retinoid X receptor (RXR) family is activated exclusively by 9cisRA [40]. 

Isotretinoin (also known as 13cisRA), an FDA approved treatment for severe acne, was reported to repress the pituitary hormones, ACTH, PRL, GH, TSH, and LH as well as cortisol during a 3-month treatment period (0.5–1 mg/kg/day) [41]. This promoted interest in the use of 13cisRA as a treatment for pituitary corticotroph tumor which is also known as Cushing Disease (CD). One study demonstrated that long-term isotretinoin treatment (60–80 mg/day for 6–12 months) led to normalization of urine free cortisol (UFC) and midnight salivary cortisol (MSC) in 6 of 16 subjects with CD (37.5%), with improved clinical symptoms and lower plasma ACTH levels [plasma ACTH (pg/mL), responders vs. non-responders, 54 ± 14 vs. 62 ± 17, *p* < 0.01] in responsive patients [42]. Tretinoin (also known as ATRA) has also been investigated in a pilot study of 7 patients with CD (3 males and 4 post-menopausal females) [43]. Five of seven (71%) exhibited reduced UFC excretion (22–73% of baseline values), with normalized UFC excretion seen in 3 patients [43]. Clinical symptoms, such as blood pressure, glycemia, and signs of hypercortisolism also improved to a variable extent [43]. Treatment with 9cisRA (2 mg/kg/day for 180 days) in 22 dogs with CD led to a significant reduction in plasma ACTH and α-MSH at 90 days of treatment compared to baseline values (*p* < 0.01), and reduced corticotroph tumor size at 180 days with improved clinical signs and increased survival time [44]. 

Mechanistic studies revealed that ATRA inhibited POMC transactivation and ACTH secretion by disrupting AP-1 and Nur77/Nurr1 transcriptional activities in murine corticotroph tumor AtT20 cells, and mutation of the Nur77/Nurr1–binding site Nur-RE abrogated the ATRA inhibitory effect [45] (Table 1). It was also demonstrated that COUP-TFα, which inhibits the POMC response to RA [46], was only expressed in normal ACTH-secreting pituitary cells but not in corticotroph tumor cells, potentially enhancing the ATRA actions in CD [45]. ATRA also induces bone morphogenetic protein-4 (BMP-4), a member of the transforming growth factor beta (TGFβ) superfamily of multifunctional secretory peptides. BMP-4 binds the activin receptor-like kinases (ALKs) to recruit and activate a cascade of transcription factors named SMADs. In AtT20 cells BMP-4 activated SMAD1 which interfered with the transcriptional activities of Pitx and Tpit through protein:protein interactions to decrease POMC transcription [47].

One potential concern of chronic isotretinoin use is that treatment has been associated with psychiatric disorders, including depression, bipolar disorder, and anxiety [48]. This situation is further complicated by the presence of three distinct RAR isoforms that have redundant but also isotype-specific activities in a context-dependent manner [49,50]. In the absence of ligand, these retinoid receptors function as trans-repressors by binding to RARE on target genes to subsequently recruit nuclear co-repressors, that include the nuclear receptor co-repressor (NCoR) and the silencing mediator of RAR and thyroid hormone receptor (SMRT). On ligand binding, RARs disassociate from their co-repressors and recruit transcriptional co-activators such as the steroid receptor co-activator-1 (SRC-1) to regulate a diverse range of target genes [51]. Comparison of these isoforms revealed variability in three residues in their ligand binding pocket which may account for the known RAR type selectivity of certain synthetic retinoids [52]. In support of this concept, the synthetic RARα/β agonist Am80 up-regulated POMC gene transcription by increasing NeuroD1 and Tpit expression [53] (Table 1). These apparent contradictory actions of RAR activation to either stimulate or inhibit POMC transcription could be explained by involvement of an RXR homodimer or permissive heterodimer that mediates these complex and mixed RXR/RAR actions [54,55]. 

#### 3.1.3. Peroxisome Proliferator Activated Receptors (PPARα, PPARβ and PPARγ)

Peroxisome proliferator-activated receptors (PPARs) are major regulators of lipid and glucose metabolism, inflammation, angiogenesis and reproduction [56,57]. By forming a heterodimer with RXR, the PPAR/RXR complex binds to a specific PPAR-responsive element (PPRE), which consists of two AGG TCA repeats separated by one nucleotide (DR1, 5′-AGG TCA N AGG TCA-3′) [58]. PPARα is highly expressed in brown adipose tissue, liver, kidney, heart, and skeletal muscle, and regulates fatty acid (FA) oxidation and transport in addition to lipoprotein synthesis in these tissues. PPARγ is mainly expressed in adipose tissue and, to a lesser extent, in the colon, the immune system, and the retina, where it regulates adipocyte differentiation and triglyceride storage. PPARβ is ubiquitously expressed and regulates lipid metabolism [59]. Similar to other NRs, in the absence of a ligand, co-repressors and histone deacetylases (HDAC) bind to PPARs and inhibit transcriptional activation of target genes. Upon ligand binding, PPARs dissociate from their corepressor complexes and recruit co-activators in order to initiate and activate transcription [60]. X-ray crystal structure analysis of PPARs revealed a much larger ligand binding pocket (1,300Å) which may explain the ability of PPARs to bind a variety of lipophilic acids [61]. Naturally occurring ligands of all three PPARs include monounsaturated fatty acids (MUFA, such as oleic acid), and polyunsaturated fatty acids (PUFA, such as linoleic acid, linolenic acid, and arachidonic acid). Additionally several P450 catalyzed arachidonic acid metabolites, including 20-hydroxy-eicosa-tetraenoic acid (20-HETE), 11, 12-epoxy-eicosa-trienoic acid (11, 12-EET), leukotriene B4 (LTB4), 9- and 13-hydroxyoctadedienoic acid (9-HODE and 13-HODE) and 15-deoxy-Δ12,14-prostaglandin J2 (PGJ2) selectively activate PPARα and PPARγ [62]. Finally, some oxidized low density lipoproteins (LDLs), alkyphospholipids, nitrolinolenic acid, and prostaglandin metabolites can selectively activate PPARγ. PPARβ can be activated by different types of eicosanoids including prostaglandin A1 (PGA1) and prostaglandin D2 (PGD2) [63]. Several synthetic PPAR ligands have been developed for therapeutic treatment of metabolic diseases, such as dyslipidemia, insulin resistance and type 2 diabetes. These drugs include fibrates, prostaglandin 12 analogs, and pirinixic acid (Wy-14643) for PPARα; hypolipidemic and hypoglycemic agents nonthiazolidinedione for PPARβ; and thiazolidinediones (TZD) such as rosiglitazone, and troglitazone for PPARγ [60]. 

All three PPAR isotypes have been detected in the normal mouse pituitary gland [64]. PPARγ mRNA and protein has been detected in normal human pituitary [65,66] and we previously demonstrated increased expression in corticotroph tumor [67] and gonadotroph pituitary tumors [68]. In mouse corticotropic pituitary tumor (AtT20) cells, the TZD compound rosiglitazone induced G_0_/G_1_ cell-cycle arrest and dose-dependently stimulated apoptosis via p53 and the mitochondrial proteins, Bcl-2/Bax [67]. In addition to its anti-proliferative effect, rosiglitazone inhibited POMC transcription and ACTH secretion in both primary human corticotroph tumor cultures and AtT20 cells [67]. Administration of high doses of rosiglitazone (150 mg/kg/day) to mice harboring corticotroph tumor delayed tumor growth and caused a 75% reduction in plasma ACTH and 96% reduction in serum corticosterone levels [67]. In a small number of patients with CD, rosiglitazone (8–24 mg/day for 1–12 months) normalized urinary free cortisol in ~30% of treated patients [69,70,71] (Table 1). However further development of this concept was hampered by occurrence of cardiac and bone toxicity in patients with diabetes treated with rosiglitazone [72], though recently a novel PPARγ agonist, MEKT1, was reported to similarly suppress POMC transcription in preclinical models of CD [73]. 

#### 3.1.4. Reverse Erb Receptors (REV-ERBα and REV-ERβ)

REV-ERBα and REV-ERBβ are the main regulators of the circadian clock system and integral in diverse metabolic processes such as adipocyte differentiation and lipid homeostasis [74]. Circadian rhythm is a ubiquitous physiological and behavioral oscillation of living organisms, entrained by an endogenous, autonomous clock system [75]. In mammals, the superchiasmatic nucleus (SCN) of the anterior hypothalamus is the master central circadian pacemaker, which senses photic information from the eyes via the retino-hypothalamic tract and communicates with peripheral tissue clocks to synchronize systemic activities via neuronal and/or hormonal signals [76]. In tandem, a peripheral clock system located in all organs and tissues including the central nervous system outside the SCN, serves as a slave clock under the regulation of the central SCN oscillator [76]. This peripheral circadian clock system comprises several interlocked positive and negative transcription/translation feedback loops coordinated by a number of self-oscillating transcription factors, such as the circadian locomotor output cycle kaput (CLOCK) and its heterodimer partner brain-muscle-arnt-like protein (BMAL1) [75]. During daytime, the CLOCK/BMAL1 complex binds to E-box elements in cis-regulatory regions of target genes to activate the principal negative regulators, period circadian regulator-1, 2 and 3 (PER-1, -2 and -3) and cryptochrome-1 and 2 (CRY-1 and -2). PER/CRY heterodimers translocate to the nucleus and interact with the CLOCK/BMAL1 complex, thereby inhibiting their own transcription [75]. During night-time, the PER/CRY complex undergoes post-translational modification mediated protein degradation, thereby releasing the negative feedback control on CLOCK/BMAL1 to activate a new cycle of transactivation, based on a 24 h interval [75]. In addition to this core loop, CLOCK/BMAL1 also controls the transcription of REV-ERBs, RORs, and E4BP4 as well as Timeless, Dec1 and Dec2, forming an auxiliary loop to modulate BMAL1 mRNA levels by competitive actions on the RER element in the BMAL1 promoter region in a 24 h cycle [75]. 

REV-ERBs don’t contain AF-2 region, and usually function as transcriptional repressors by recruiting silencing complexes such as NCoR-HDAC3 directly to the promoter regions of target genes [77]. REV-ERBs bind to the same RORE DNA response elements as RORs in the form of both a monomer (an AGG TCA half site with a 5′AT-rich extension) or a homodimer (RevDR2, RGG TCA NN RGG TCA) [78]. This interactive positive and negative regulation on RORE transactivation by RORs and REV-ERBs shapes the oscillating expression of BMAL1, while REV-ERB expression is negatively regulated by PER/CRY proteins and positively regulated by CLOCK/BMAL1, complicating the feedback loop of the circadian clock system [79]. REV-ERBα null mice display altered circadian rhythms characterized by increased CLOCK/BMAL1 expression and shorter period lengths when compared to wild-type mice [78]. These REV-ERBα null mice also exhibited a 2.5 fold increase in adiposity and mild hyperglycemia due to altered lipid and glucose metabolism [80]. 

The pituitary gland participates in SCN control of peripheral clocks by its pulsatile secretion of pituitary hormones into the bloodstream [81]. The hypothalamic PVN contains parvocellular CRH and AVP neurons that project downwards to the median eminence (ME) of the hypothalamus to release CRH and AVP into the hypothalamic-pituitary portal circulation [82] (Figure 1B). By receiving these efferent connections from the SCN, the PVN acts as a node to translate central diurnal oscillation into HPA axis circadian rhythmicity by regulating hypothalamic CRH and subsequently pituitary ACTH release to cause diurnal serum cortisol fluctuations [83]. In addition, the SCN projects to the autonomic nerve system (ANS) from which splanchnic nerve innervation connects the adrenal medulla to the central circadian pacemaker, regulating adrenal sensitivity to ACTH by the action of the sympathetic hormone epinephrine and other adrenal medulla secretory products [76].

GCs are hypothesized to inhibit stimulation of the CRH-dependent circadian rhythm by a negative feedback loop at both pituitary and hypothalamic levels [84]. However, the daily expression pattern of PER-2 and BMAL1 in the pituitary gland of adrenalectomized mice was quite similar to that observed in sham-operated animals, suggesting that the intrinsic self-sustaining circadian clock system of the pituitary is quite resilient to GC regulation [85]. In line with this, pituitary explants from the PER-1-Luc transgenic rat, where luciferase expression was driven by the PER-1 gene promoter, exhibited circadian rhythmicity that persisted for multiple days during in vitro cultures [86]. This kind of self-sustained circadian rhythm generation of bioluminesce was also observed in pituitary explants from PER-2-Luc [87] and BMAL1-Luc [88] transgenic mice. As further evidence of the importance of the pituitary clock, circadian expression of pituitary POMC and PRL mRNA was observed in cultured murine pituitary tumor cell lines (AtT20, GH3 and GH4C1), and their expression correlated to the circadian proteins (PER-2 for POMC, and CLOCK for PRL) [89,90]. Detection of clock gene expression in human autopsy pituitary tissues revealed that only PER-1 showed daytime-dependent differences in quantitative real-time PCR (qPCR) analyses [91], indicating that pituitary hormone synthesis exhibits significant adaptive plasticity to central and intrinsic temporal modulatory signals. 

#### 3.1.5. Retinoic Acid Receptor-Related Orphan Receptors (RORα, RORβ and RORγ)

The retinoic acid receptor-related orphan receptor (ROR) family, discovered in 1994, plays important roles in metabolism, development, immunity and circadian rhythm [92,93,94]. RORs bind preferentially as monomers to an ROR response element (RORE) composed of a hexanucleotide motif with an upstream AT-rich sequence (typically consisting of TAAA/TNT AGG TCA) on the promoter of target genes [95] and their transactivation is more potent when they form a homodimer on a RevDR2 (RGG TCA NN RGG TCA) [96]. In contrast to REV-ERBs, RORs often function as transcriptional activators and their expression correlates with histone acetylation and chromatin accessibility [97]. RORs have a tissue specific distribution, with RORβ restricted to the brain and RORγ mainly in skeletal muscle [98]. RORα is broadly distributed in many peripheral tissues, including liver, smooth muscle, testes, and lymphocytes [99]. 

Spontaneous mutations of RORα have been found in both mice [100] and humans [101,102]. The RORα mutant mouse, named “staggerer”, harbors a truncated RORα1 that lacks the entire LBD due to a spontaneous 6.5kb genomic deletion on Chr9, which introduces a premature stop codon in Exon3 [103]. Staggerer mice exhibit defects in brain development [104,105], lipid metabolism [106] and inflammatory response [107]. They also exhibit disrupted circadian patterns with shortened period length when kept in constant dark conditions due to reduced expression of BMAL1 [108]. RORα and RORβ are weakly expressed in normal mouse and ovine pituitary gland [109,110]. Staggerer mice exhibit an enhanced corticosterone and ACTH response to novel environmental stimuli and lack diurnal variation in corticosterone secretion compared to wild-type mice [111]. However, a potential direct action of RORα on pituitary ACTH hormone production needs further validation since global RORα deletion with its effect on HPA feedback makes it difficult to interpret the ACTH response in the “staggerer” RORα deletion mice. 

#### 3.1.6. Liver X Receptors (LXRα and LXRβ)

The liver X receptors (LXRs) are known as oxysterol sensors, and regulate a variety of genes involved in cholesterol and lipid metabolism [112,113]. LXRα is most highly expressed in the liver and to a lesser extent the kidney, small intestine, spleen, and adrenal gland; whereas LXRα is ubiquitously expressed [114]. LXRα and LXRβ form obligate heterodimers with RXR, and the LXR/RXR heterodimer is a permissive heterodimer that can be activated by either an LXR agonist or the RXR ligand 9cisRA [115]. The LXR response element (LXRE) consists of two hexanucleotide repeats (5′-AGG TCA-3′) separated by four or one nucleotide(s) (DR4 or DR1) [115]. LXR target genes are involved in reverse cholesterol transport [such as ATP-binding cassette transporters A1 (ABCA1) and G1(ABCG1)], cholesterol conversion to bile acid (SREBP-1c), intestinal cholesterol absorption [apolipoprotein E (apoE)], and lipogenesis [FAS and stearoyl-CoA desaturase (SCD)] [116]. Endogenous ligands of LXRs include the oxidized cholesterol derivatives 20(S)-, 22(R)-, 24(S)-, 25- and 27-hydroxy cholesterol and 24(S), 25-epoxycholesterols [117]; and endogenous antagonists include arachidonic acid and its cyclooxygenase metabolites, such as PGF_2α_, ursodeoxycholic acid and 5,6-epoxycholesterol [118]. TO901317 is a non-steroidal synthetic ligand of LXR that induces ABCA1 expression [116], while it is also an effective activator of the pregnane X receptor (PXR) and the farnesoid X receptor (FXR), as well as an inhibitor of RORs [77]. 

Using qPCR, it has been demonstrated that LXRβ is expressed throughout brain, and LXRα is specifically expressed in the pituitary and particularly in the anterior lobe of the pituitary [119]. Both LXRα and LXRβ are expressed at lower levels in human pituitary adenomas (*n* = 23) compared to normal pituitary tissues (*n* = 3) [120], where it was also observed that LXRα was more highly expressed than LXRα in ACTH-secreting pituitary adenomas. Other studies showed that the LXR agonist TO901317 increased POMC expression and ACTH secretion in vitro and in vivo [119,121], which may in part be due to an action of TO901317 to inhibit 11β-hydroxysteroid dehydrogenase type 1 (11β-HSD1), a key enzyme that catalyzes the intracellular conversion of cortisone to physiologically active cortisol. By this manner, TO901317 reduced local cortisol levels, resulting in increased ACTH levels due to reduced glucocorticoid negative feedback [121]. In murine corticotroph tumor cells, direct binding of an LXR/RXR heterodimer to a POMC promoter DR4 motif (-73 AGGAAGGTCA CGTC CAAGGCTCA -52) was demonstrated, where LXRα specifically induced POMC transcription [119] (Table 1). Although LXR null mice (LXRα/β^−/−^) exhibited an elevated circulating ACTH level, pituitary POMC mRNA expression largely remained unchanged. These discrepancies suggest that LXR may regulate the HPA axis at multiple levels, or possibly that TO901317 exerts effects mediated by other NRs such as ROR and/or PXR. 

#### 3.1.7. Pregnane X Receptor (PXR) and Constitutive Androstane Receptor (CAR)

Pregnane X receptor (PXR) and constitutive androstane receptor (CAR) are two closely related liver-enriched NRs that play important roles in xenobiotic metabolism [122,123,124]. They function as endobiotic sensors for toxic byproducts derived from glucose and lipid metabolism and act to enhance their elimination in the liver and intestine [125]. PXR and CAR bind as a heterodimer with RXR to a xenobiotic response element (XRE) on the promoter of target genes. The XRE motif comprises two copies of the half site consensus sequence AG(G/T)TCA with various spacing, including DR-3, 4, and 5, and everted repeats (ER-6 and 8) [126]. PXR has many endogenous ligands, including the steroid hormones, 5β-pregnane-3,20-dione, progesterone, corticosterone, testosterone, pregnenolones, lithocholic acids, the steroid-like compound dexamethasone, and 17β-estradiol [127]. PXR pharmacological ligands include the antibiotic and antifungals, rifampicin and clotrimazole; the anti-cancer agents, tamoxifen and taxol; the anti-hypertensive nifedipine; and anti-depressant hyperforin [128]. Structural analysis reveals that PXR contains an expansive ligand binding pocket capable of changing in shape depending on the nature of its bound ligand [129]. The PXR LBD sequence varies significantly across species, suggesting that PXRs have evolved according to various xenobiotic or endobiotic pressures unique to each species [130]. 

PXR plays an important role in adrenal steroid homeostasis [131]. Transgenic mice expressing a constitutively active form of human PXR (VP-hPXR) exhibited markedly increased plasma and urinary concentration of corticosterone [132]. Activation of PXR in transgenic mice increased expression of the adrenal steroidogenic enzymes, CYP11a1, CYP11b1, CYP11b2, and 3β-HSD [133]. However, plasma ACTH level was normal in the PXR transgenic mice, and they exhibited normal corticosterone suppression in response to dexamethasone, indicating that PXR activation does not interfere with the HPA axis function in spite of severely disrupted adrenal steroid homeostasis [133].

### 3.2. Subfamily 2

This subfamily contains 12 members belonging to 5 groups. They include hepatocyte nuclear factors (NR2A1 and NR2A2), retinoid X receptors (NR2B1, NR2B2, and NR2B3), testicular orphan nuclear receptors (NR2C1 and NR2C2), tailless homolog orphan receptor (NR2E1) and photoreceptor-cell-specific nuclear receptor (NR2E3), chicken ovalbumin upstream promoter-transcription factors (NR2F1, NR2F2 and NF2F6). Except for NR2As and NR2Bs, most members of this subfamily remain orphaned.

#### 3.2.1. Retinoid X Receptors (RXRα, RXRβ, and RXRγ)

RA exerts its biological activities through complicated mechanisms depending on the amount and ratio of tissue-specific metabolite isomers [53]. The retinoic acid receptor (RARα, RARβ, RARγ) and retinoid X receptor (RXRα,RARβ, RXRγ) are the cogent nuclear receptors of retinoic acids [51]. 9cisRA was the first natural ligand identified for RXRs [54] and further studies revealed that as RXRs also heterodimerize with other nuclear receptors, they are activated by a wide spectrum of ligands [54]. At the molecular level, when RXRs homodimerize or heterodimerize with PPARs, LXRs and PXR, the receptor complex responds to agonists of both RXRs and their partners, and these types of heterodimers are called permissive heterodimers [134]. Conversely, when RXR forms a heterodimer with the TRs, RARs, or VDR, the complex is only activated by the partner agonist but not by an RXR ligand, generating a group of so-called non-permissive heterodimers [55]. Similar to the effects of ATRA [45], the synthetic RXR selective pan agonist HX630 was shown to negatively regulate POMC transcription by suppression of Nur77 and Nurr1 expression, which may be mediated through either an RXR homodimer or a permissive RXR heterodimer [55] (Table 1). Given PPARγ was the only permissive RXR partner related to POMC trans-repression [67,135,136], the RXR/PPARγ heterodimer was suspected to mediate the anti-proliferative and POMC inhibitory effects of HX630, raising the possibility that a combination of an RXR agonist with a PPARγ agonist could be a potent regulator of POMC transcription. 

#### 3.2.2. Testicular Orphan Nuclear Receptor-4 (TR4)

Testicular orphan nuclear receptor-4 (TR4) was cloned from human testis cDNA libraries [137] and plays key roles in aging, cancer, metabolic diseases and central nervous system development [138,139,140]. TR4 knockout mice exhibit growth retardation and defects in reproduction and maternal behavior as well as deficits in spermatogenesis and motor coordination with aberrant cerebellar development [141]. TR4 binds to an AGG TCA motif with a variable number of spacer nucleotides (DR-0 to 6) in target gene promoters [142]. TR4 has been shown to repress RXR/RAR-mediated transactivation by competitively occupying DNA responsive elements of RXR-RE (DR1) and RAR-RE (DR5), without affecting protein-protein interaction [143]. As part of a feedback mechanism, TR4 expression itself is induced by retinoic acid [143]. On the other hand, TR4 mediates the pro-apoptotic and differentiation inducting effect of retinoic acid in mouse p19 teratocarcinoma cells, a model used to study neuronal development [144]. Blockage of TR4 actions in p19 cells generated resistance to retinoic acid-induced cell death, and may in part explain the impaired cerebellar development in TR4 null animals [145]. 

Our recent studies demonstrated that TR4 functions as an important modulator of HPA axis homeostasis by positively regulating POMC gene transcription [146,147]. Using a genetic (siRNA and shRNA directed knockdown) approach to abrogate TR4 expression and small molecule inhibitor (MEK-162) to block TR4 post-translational activation, we demonstrated that TR4 regulates ACTH secretion and tumor growth in both in vitro and in vivo models of CD [146,147,148] (Table 1). We further demonstrated that TR4 directly interacts with the GR to override the negative actions of GR to repress POMC transcription [147]. 

#### 3.2.3. Chicken Ovalbumin Upstream Promoter Transcription Factors (COUP-TFα, COUP-TFβ and COUP-TFγ)

Chicken ovalbumin upstream promoter transcription factors (COUP-TFs) are highly conserved across species [149] and bind promiscuously to a wide range of direct GGTCA repeat with different spacing and orientations, repressing gene expression either by active repression or by competition for binding sites of other transcription factor [46,150]. COUP-TFα is highly expressed in neuronal tissues of the central and peripheral nervous systems, whereas COUP-TFβ is mainly expressed during embryonic development to regulate neural, muscular, bone, liver and skin differentiation [151]. COU-TFs have lineage-restricted distribution in the normal pituitary, with COUP-TFα expressed exclusively in normal corticotrophs, whereas COUP-TFβ and COUP-TFγ are expressed predominantly in lactotrophs [149]. In contrast, human corticotroph tumors did not express COUP-TFα, suggesting a possible role for COUP-TFα in dysregulated corticotroph cells [45]. 

### 3.3. Subfamily 3

This subfamily comprises 3 groups and 9 steroid receptors, namely NR3A1 (estrogen receptor-α, ERα), NR3A2 (estrogen receptor-β, ERβ), NR3B1 (estrogen related receptor-α, ERRα), NR3B2 (estrogen related receptor-β, ERRβ), NR3B3 (estrogen related receptor-γ, ERRγ), NR3C1 (glucocorticoid receptor, GR), NR3C2 (mineralocorticoid receptor, MR), NR3C3 (progesterone receptor, PR) and NR3C4 (androgen receptor, AR). Other than the ERRs which are without known ligands (orphan receptors), the other steroid receptors in this group are activated by cholesterol-derived hormones and then translocate to the nucleus to modulate downstream target expression. 

#### 3.3.1. Estrogen Receptors (ERα and ERβ)

**Figure 5:**
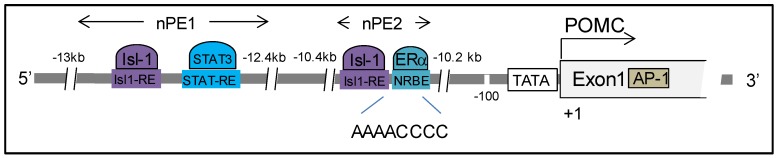
**Illustration of hypothalamic POMC transcriptional regulation.** Distinct from the pituitary POMC promoter, two target sequences nPE1 and nPE2 on the POMC enhancer contribute to hypothalamic POMC neuron transcriptional regulation. AP-1, activator protein-1; Isl-1, ISL LIM homeobox 1; Isl1-RE, Isl-1 responsive element; NRBE, nuclear receptor binding element; STAT3, signal transducer and activator of transcription 3; STAT-RE, STAT responsive element.

The hypothalamic POMCergic system plays a key role in energy homeostasis and pain sensitivity, and neuronal POMC transcription and peptide processing is regulated by a series of factors distinct from those in the pituitary. Instead of the 5′ proximal −480 bp anterior pituitary corticotroph promoter, two enhancer regions located 10-12kb upstream from the transcription initiation site (nPE1 and nPE2) contribute to hypothalamic POMC transcriptional regulation (Figure 5). Using a highly conserved half-site nuclear receptor binding element (NRBE, AAA ACC CC) present in nPE2, de Souza et al. performed yeast one-hybrid screening of an adult mouse brain cDNA library and identified ERα (also known as ESR1) as a potential transcriptional regulator of hypothalamic POMC, suggesting that estrogen exerts its anorectic effect by controlling neuronal POMC expression [152] (Table 1). 

ERα and ERβ (also known as ESR2) share 95% amino acid sequence homology in their DBD, and 55% homology in their LBD [153]. ERα immuno-reactivity in the normal human pituitary gland is most highly expressed in gonadotrophs (FSH+ 70%, LH+ 83%) followed by lactotrophs (50%), thyrotrophs (4%), and corticotrophs (1%) (154). In human pituitary adenomas, ER is highly expressed in prolactinomas [154,155,156,157]; while ACTH adenomas and silent coriticotroph adenomas were the least immune-reactive for both estrogen receptors [158]. ER is known to activate PRL transcription by directly binding the PRL gene distal enhancer, which synergizes with Pit-1 [159,160,161,162]. Disruption of murine ERα by a targeted insertion reduces anterior pituitary gland size and leads to decreased lactotroph cell number and a marked reduction in PRL mRNA [163]. POMC, TSHβ, αGSU, LHβ and FSHβ subunit mRNA levels were all increased in ERα gene-disrupted mice and accompanied a relative increase in density of these cell subtypes [163]. Interestingly, multiple tumor-specific ER splicing variants have been found in pituitary tumors with expression of Δ5ERα in 100% of null cell tumors, 80% of somatotroph tumors and 60% of corticotroph tumors [164]. However, the role, in any, of these ER variants in pituitary tumor generally and corticotroph tumor specifically awaits more detailed studies.

#### 3.3.2. Glucocorticoid Receptor (GR)

**Figure 6:**
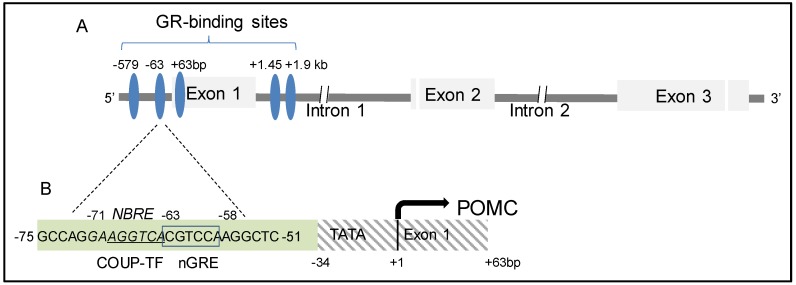
**Delineation of the target regions of the pituitary POMC promoter regulated by the glucocorticoid receptor.** (**A**) Five GR binding segments (blue circles) have been characterized as the negative glucocorticoid responsive elements (nGREs) of the POMC gene. (**B**) By deletion and mutation of POMC promoter regions, a hexanucleotide (-63 CGTCCA -58, boxed sequence) was demonstrated to act as a nGRE. A COUP-TF binding site (-69 AGGTCA -64, underlined sequence), and a naturally occurring cis-acting Nur77-binding response element (NBRE, -71 GAAGGTCA -63, Italics sequence) are located immediately adjacent to the nGRE.

Glucocorticoids (GCs) regulate a wide variety of biological processes involved in development, growth, metabolic homeostasis, inflammation, and cognition. These are mediated through the multifaceted glucocorticoid receptor (GR) [165]. GCs negatively regulate HPA axis activity by directly targeting both hypothalamic CRH and pituitary ACTH synthesis and secretion respectively [166]. To characterize the negative glucocorticoid responsive element (nGRE) of the POMC gene, Drouin et al. performed DNAse-I and exonuclease-III footprint experiments and demonstrated 5 GR binding segments, 2 localized in the 5′-flanking region (centered on position −579 and −63 bp), 1 in Exon-1 (+63 bp), and 2 in Intron-1 (+1.45 and +1.9 kb) [167,168] (Figure 6A, blue circles). Following additional deletion and mutation of the selected POMC promoter regions, they further demonstrated that a hexanucleotide (-63 CGT CCA -58, boxed sequence, Figure 6B), similar to the GRE consensus TGT YCT, acted as a negative glucocorticoid regulatory element (nGRE) [169]. Differing from the conventional GR homodimer that binds to the positive regulatory GRE, 3 GR-ligand complexes bind to the POMC nGRE through the sequential binding of a GR homodimer followed by binding of a GR monomer on the opposite side of the DNA double helix to enhance affinity [170]. Although the nGRE is adjacent to the COUP-TF binding site (-69 AGG TCA -64, underlined sequence, Figure 6B), competition between GR and COUP-TF was excluded by negative binding of a COUP-TF antibody to the nGRE, and COUP oligonucleotide probe to the GR antibody in EMSA assays [170] (Table 1). 

The human GR gene locus maps to the long arm of chromosome 5 (5q31.3), spanning 160 kb on the reverse strand, and gives rise to several diverse GR variants [171]. The human GR promoter does not have a classic TATA box or CCAAT motif as transcription initiation sites, so depending on tissue-specific promoter usage, GR transcripts have 13 alternative variants for Exon 1 generating a 5′-UTR of various lengths [172]. GRα is the dominant active GR form composed of 777aa (transcripts containing Exon 2–8 plus 9α), and contains 5 major structural domains, namely an N-terminal transactivation domain (NTD, 1–420aa), a DNA binding domain (DBD, 421-485aa), a hinge domain (H, 486–527aa), a ligand binding domain (LBD, 528–726) and a C-terminal transactivation domain (CTD, 727–777aa) [173]. Upon ligand binding, GRα undergoes a conformational change, dissociates from its cytoplasmic chaperones and translocates into the nucleus to regulate downstream gene transcription in a genomic direct or indirect-manner [174]. GRβ is the second GR isoform that results from an alternative splicing acceptor site in Exon 9 downstream (transcripts containing Exon2–8 plus 9β), and differs from GRα in the CTD by replacement of the last 50 aa (727–777aa) with 15 non-homologous sequences (727–742aa) [175]. GRβ functions as a dominant negative regulator of GRα, does not bind glucocorticoids, but constitutively localizes in the nuclei. There it competes with GRα to bind GRE consensus sites and transcriptional co-regulators, and disrupts GRα homodimerization [171]. Although GRβ is generally expressed at lower levels than GRα, increased GRβ levels have been observed in a variety of inflammatory diseases and is associated with resistance to the actions of glucocorticoids (GC resistance) [171]. GRγ is the third GR variant and differs from GRα by one arginine (R) residue at the 452nd position in the DBD due to use of an alternative splice donor site in Intron 5 [176]. This extra R452 insertion is located between the two C4-type zinc finger structures of the GRγ DBD, and alters its transcription profile without affecting cellular distribution or protein partner binding [171]. GRγ expression is associated with GC resistance in small cell lung cancer, corticotroph adenomas [177] and childhood leukemia [171]. Two additional GR splicing variants, GR-A and GR-P have large structural truncations in their LBD [165]. GR-A contains 592aa, and lacks the amino-terminal half of the LBD (deletion of Exon 5–7, A490-S674) due to an in-frame juxtaposition of Exon 4 and Exon 8 [178]. GR-P contains 676aa (Exon 2–7) and results from a premature stop due to splice failure at the Exon7-8 boundary near the LBD C-terminus [165]. Upregulation of GR-P has been observed in several GC-resistant hematological malignancies [174]. Alternative translational initiation sites caused by leaky ribosomal scanning or ribosomal shunting mechanisms produce 8 more GR subtypes (A, B, C1–C3, D1–D3) with progressively shorter NTDs. A ligand-independent constitutively active function domain (AF-1, 77–262aa) in the NTD is key for recruitment of transcriptional coregulatory partners, therefore obstruction of the AF-1 in GR-C and GR-D isoforms leads to differential GR cellular compartmentation, gene regulatory profiles, and GC responses although they share similar ligand affinity and DNA binding capability [171]. For instance, GRα-C2 and –C3 isoforms have stronger trans-activation than GRα-D isoforms even though the latter constitutively reside in the nucleus [179]. Therefore tissue-specific translational isoform alterations in various diseases partially explain some observed mechanisms of GC resistance.

Sporadic GR mutations characterized by hypercortisolism without Cushingoid features further reveal the importance of the GR in generalized GC resistance [180]. Seventeen natural GR mutations in more than 20 kindreds and sporadic cases, associated with altered GR expression, ligand affinity, GR protein turnover, and cellular translocation have been described [181]. Among the 12 pathological mutations in the LBD region, 8 (T556I, I559N, G679S, R714Q, H726R, F737L, I747M, L773P) were heterozygotic mutations indicating that complete loss-of-function of these residues is incompatible with life [182]. The homozygotic mutations of V571A, D641V, V729I and F774 frameshift cause a dramatic reduction in ligand affinity (from complete absence of ligand-binding activity with F774FS to a 3-fold reduction with D641V) [182]. Delay in nuclear translocation of the GR mutants was observed following Dexamethasone treatment, ranging from 20 min with R714Q to 180 min with I559N [179] and they all exhibited an abnormal interactions with the transcriptional coregulator glutamate receptor interacting protein 1 (GRIP1), resulting in reduced transcriptional activation [179]. In contrast, the D401H mutation located in the NTD exhibited normal ligand affinity and nuclear translocation with increased transactivation [179]. Human phenotypes associated with the D401H mutant include visceral obesity and type 2 diabetes, which are quite distinct from the LBD mutants which are associated with hirsutism, fatigue and hypoglycemia [183]. Three heterozygotic mutations in the DBD include two point mutations (V423A and R477H) with reduced transactivation and one non-functional truncation (R469*) which causes 50% reduction of GR protein expression due to nonsense-mediated mRNA decay [181]. Additional 616 GR single nucleotide polymorphisms (SNPs) have been reported for GR (https://www.ncbi.nlm.nih.gov/SNP/snp_ref.cgi?locusId=2908). Among these SNPs, ER22/23EK affects the expression of the GRα-A translational isoform causing relative GC resistance and resulting in greater insulin sensitivity and lower low-density lipoprotein cholesterol levels [184]. The N363S SNP mildly increases GC sensitivity and correlates with central obesity [185]; whereas a silenced SNP of A3669G in Exon 9β 3′-end increases GRβ mRNA stability leading to greater inhibition of GRα-induced transcriptional activity and causing GC resistance [186]. 

In addition to the natural occurring mutations and polymorphisms, reversible post-translational modifications may also contribute to GR functional regulation [187]. Most GR phosphorylation sites are identified in the NTD region, among which phosphorylation of S203, S211, S226 and S404 are the best characterized [188]. Several CDK complexes (CyclinA/CDK2, Cyclin B/CDK2, Cyclin E/CDK2 and CDK5) and ERK can phosphorylate GR S203, which leads to GR cytoplasmic retention and transcriptional inactivation [20]. GR S226 phosphorylation by JNK and CDK5 also blunt GC signaling by enhancing GR nuclear export [188]. GR S404 phosphorylation by GSK3β affects its association with the transcription factor p65 (NFκB subunit) and the transcriptional coactivator p300/CBP [188]. S211 phosphorylation by the CDK complex and p38-MAPK generates an active form of GR that mediates GC-induced apoptosis in lymphoid cells [188]. Two MAPK components, JNK and ERK, inhibit S211 phosphorylation and attenuate GC sensitivity, indicating that different phosphorylation patterns induced by various upstream effectors serve to direct the cellular receptors to GCs [188]. Lysine426 is the ubiquitin acceptor site of the GR and several RING-type E3 ligases (HDM2, CHIP, FBXW7 and UBR1) contribute to substrate recognition and poly-ubiquitin chain conjugation, leading to proteasome-dependent GR protein degradation following ligand binding [189]. SUMO (small ubiquitin-related modifier) is an ~100 aa peptide that can be catalyzed to modify substrate via a cascade of reactions involving the E1 activation enzyme, the E2 conjugation enzyme and the E3 recognition ligase [178]. GR has three SUMO acceptor sites, two suppressive modification sites in its NTD (K277 and K293) and one active site in its LBD (K703) [190,191]. 

### 3.4. Subfamily 4

The Nur subfamily comprises three NR members, namely NR4A1 (also known as nerve growth factor induced clone B (NGFI-B), Nur77), NR4A2 (Nurr1) and NR4A3 (NOR-1) [192]. The Nur receptors were first identified as immediate-early response genes induced by nerve growth factors in rat pheochromocytoma PC12 cells [193]. Their expression is rapidly and transiently induced in the central nervous systems in response to physiological, chemical or toxic stimuli and they are present throughout the HPA axis [194]. 

#### 3.4.1. Nerve Growth Factor Induced Clone B (NGFI-B, Nur77)

Nur77 was the first NR demonstrated to bind a DNA consensus sequence as a monomer (others bind as homo- or heterodimers). The octanucleotide of the Nur77-binding response element (NBRE) contains 5′-AAA GGT CA-3′ [195]. When Nur77 forms a homodimer or heterodimer with other Nur factors, the complex binds to a Nur-responsive element (NurRE) which contains an inverted repeat of the NBRE-related octanucleotide separated by six nucleotides. Nur77 and Nurr1 also form heterodimers with RXRs to mediate retinoid signaling, and they bind to a direct repeat 5 (DR5) motif which comprises two direct repeats of the consensus NR binding motif AGG TCA separated by five nucleotides [196]. A naturally occurring cis-acting element of NBRE is found on the proximal POMC promoter (-71 GAA GGT CA -63) located within the nGRE [192,197] (Figure 6B, Italics sequence). Although Nur77 bound to the nGRE under basal conditions in a gel shift assay [197], mutation of this NBRE did not change CRH-induced Nur77-dependent POMC transactivation [198]. In contrast, mutation of an alternate Nur-RE (-405 TGA TAT TTA CCT CCA AAT GCC A -384) which is located upstream to the tissue restricted E-box_NeuroD1/Pan1,_ T-box_Tpit_, and Pitx-RE, abrogated Nur77- and CRH-induced POMC transactivation [198] (Figure 3D). Additionally, this NBRE site was insensitive to interleukin-1 (IL-1) mediated induction of POMC transcription, although Nur77 homodimeric binding and Nur-RE-dependent activation preponderantly regulate POMC mRNA induction and ACTH secretion following IL-1 treatment [199]. This observation underlies the synergistic modulatory actions of CRH and IL-1 at the pituitary level [200,201] (Table 1). Nur-RE is also the target of GC actions through a direct protein:protein interaction between Nur77 and GR. GCs antagonize the Nur-RE response to CRH, which may explain the insensitivity of corticotroph cells to hypothalamic-derived pulsatile CRH control under chronic stress [202,203]. 

As similar to other groups of NRs, the NR4A subgroup members have a highly conserved DBD (~91–95%) and C-terminal LBD (~60%). However they have a different NTD [204], which harbors several post-translational modification sites, including phosphorylation [205] and SUMOylation [206]. In response to CRH/cAMP treatment, Nur77 is phosphorylated by extracellular signal regulated kinase (ERK) in a protein kinase A (PKA)/calmodulin kinase II (CAMKII)-dependent manner which then enhances Nur77 transcriptional activity [205]. In parallel, Nur77 S316 undergoes dephosphorylation following CRH treatment, leading to dimerization and recruitment of co-activators [198,207]. The p160 steroid receptor coactivators (SRC-1, 2, and 3) are the co-activators of Nur77 and interact with Nur77 through its NTD AF-1 region [207]. By recruitment of histone modification enzymes, such as histone acetyltransferase CBP/p300 and the histone methyltransferase CARM-1, these cofactors form a high molecular weight protein complex on target gene promoters to synergistically enhance Nur77-depedent transcription [208,209]. In addition, TIF1β [also known as KAP-1 (KRAB domain–associated protein), or KRIP-1] has been identified as a potent Nur77 coactivator via mass spectrometry from a multiprotein complex co-immunoprecipitated with Nur77 [210]. Following CRH stimulation, ITF1β was recruited to the POMC promoter by Nur77 and functioned as part of a common transcriptional complex to synergistically enhance Nur77-dependent POMC transcription [210].

Crystallographic studies have shown that the putative ligand-binding pocket of Nur77 LBD is filled with side chains of large hydrophobic amino acids [211], which keep the LBD in a transcriptionally active conformation [212]. Recently, several small molecules have been demonstrated to interact with Nur77 and modulate its transcriptional activity, including 1,1-bis(3′-indolyl)-1-(p-substitutedphenyl) methanes [213,214,215], the octaketide, cytosporone B [216], the PUFAs arachidonic acid (AA) and docosahexaenoic acid (DHA) [217] and prostaglandin A2 [218]. These synthetic and naturally occurring ligands have been demonstrated to induce Nur77-mediated protection against various processes including neuro-inflammation and neuronal cell death [219], cardiac remodeling [220], cancer cell proliferation [221] and inflammatory lung disease [222]. In addition to activation by ligands, Nur77 is under tonic inhibitory dopaminergic (DAergic) control mediated by D2 receptors in the striatum, the nucleus accumbens and the prefrontal cortex of the ventral midbrain region [194]. In support of this, D2R agonist administration decreases Nur77 mRNA levels in the striatum [223]. This inhibitory effect on Nur77-mediated POMC transcription was also observed in corticotroph tumors [224]. Nur77 is also induced in the HPA axis and adrenal medulla during the stress response [194]. However, Nur77 null mutant mice display intact function of the HPA axis, which likely reflects some functional redundancy as a compensatory increase in Nurr1 mRNA expression occurs in the adrenal gland of Nur77 null mutant mice following HPA axis stimulation [225].

#### 3.4.2. Nur-Related Factor 1 (Nurr1)

Nurr1 was cloned as a predominantly brain-specific gene whose expression pattern is similar to that of Nur77 and NOR-1 [226]. During early embryonic development, Nurr1 is selectively expressed in the diencephalic regions that give rise to the hypothalamus, whereas Nur77 is not expressed until the postnatal stage [192]. Unlike Nur77/NOR-1, Nurr1 is constitutively expressed in the hypothalamic PVN, which suggests that it participates in basal expression of tissue specific genes [227]. Meanwhile Nurr1 is the only Nur factor that is expressed in the affected DAergic neurons of the substantia nigra and in the ventral tegmental area of the midbrain [228]. 

Binding of Nurr1 to the NBRE in the rat POMC and ovine CRH promoter regions (POMC, -70/-47 within nGRE; CRH, -352/-332) was confirmed by electrophoretic mobility shift assay (EMSA) respectively, and this Nurr1-dependent NBRE activation is responsible for forskolin-induced POMC transactivation as well as GR-regulated repression of POMC promoter activity [192]. Nurr1 interacts directly with GR via the N-terminal 120aa of Nurr1, and this Nurr1-GR heterodimerization modulates Nurr1-dependent transcriptional activity [229] Nurr1 is an atypical NR due to its ligand and coactivator-independent transcriptional activity [230]. Only RXRs and PIASγ have thus far been shown to interact directly with Nurr1 to modulate its transcriptional activity [231,232]. By affinity chromatography, several kinases have been shown to interact with Nurr1 to increase (ERK2 and 5) or decrease (LIMK1) NBRE-containing promoter transcriptional activity [230]. Similar to Nur77, the Nurr1-mediated POMC Nur-RE is more responsive to CRH stimulation compared to the NBRE [205]. CRH also induces Nurr1 through the cAMP/PKA pathway to activate POMC gene expression. In response to proinflammatory cytokines, such as TNFα and IL-1β, Nurr1 expression is stimulated through NFκB-dependent transactivation, which further increases POMC expression as a central response to infection/inflammation stress [233].

Similar to PPARs, LXRs, FXR, HNF4s, RXRs, and Nur77, Nurr1 directly binds UFA (AA and DHA) at micromolar concentrations [234]. Using nuclear magnetic resonance (NMR) spectroscopy and hydrogen/deuterium exchange coupled to mass spectrometry (HDX-MS), which are sensitive to protein dynamics in solution compared to static crystallography, de Vera and colleagues demonstrated that binding of AA to Nurr1 increased the solvent exposure of the Nurr1 ligand binding pocket (LBP), indicating that UFAs can indeed bind to the canonical LBP in the Nurr1 LBD [235]. In line with NMR spectroscopy data, molecular dynamics simulations revealed an overall similar pocket location of Nurr1, and minor structural rearrangements within the LBD can create sufficient space for ligand DHA [236]. These analyses not only facilitate our understanding of interactions between Nurr1 and its known ligands, also predict other potent modulators which may affect Nurr1 activity and be used in Nurr1-targeted treatment strategies. 

#### 3.4.3. Neuron derived Orphan Receptor 1 (NOR-1)

NOR-1 and Nur77 are highly expressed in the pituitary gland with low expression in the thymus, kidney, heart, skeletal muscle, and adrenal glands [194]. Unlike Nur77 knockout mice which lack a clear phenotype, NOR-1 deficient mice exhibit abnormal ear and hippocampus development, and have a defect in gastrulation [237]. Lack of both NOR-1 and Nur77 induced catastrophic deregulation of the immune system, and led to development of fatal acute myeloid leukemia in mice, indicating that these nuclear receptors may play a role as tumor suppressors [238]. In line with this, NOR-1/Nur77 mediated the apoptosis of immature thymocytes in response to T-cell antigen receptor signaling during T cell development [239,240]. Mitochondrial translocation of Nur factors and interaction with Bcl-2 were associated with a pro-apoptotic effect of NOR-1/Nur77 [241]. 6-mercaptopurine (6-MP), a widely used anti-neoplastic and anti-inflammatory drug, functions as an agonist of NOR-1 by activating its AF-1 domain [242]. NOR-1 has comparatively little impact on the POMC promoter [227], since it can only bind the POMC Nur-RE site and activate transcription as a heterodimer together with Nur77 [194]. However, similar to other Nur factors, NOR-1 is rapidly and transiently induced after CRH treatment to activate the HPA axis with increased ACTH and cortisol release [227]. 

### 3.5. Subfamily 5 and 6

Subfamily 5 contains steroidogenic factor-1 (SF-1, NR5A1) and liver receptor homolog-1 (LRH-1, NR5A2). Phospholipids have been suggested as their possible ligands [243]. These NRs are required for differentiation of steroidogenic tissues, and are essential for pituitary gonadotroph function [244,245]. Germ cell nuclear factor (GCNF, NR6A1) is the only orphan receptor of subfamily 6, and is expressed at a very high levels in developing male germ cells and growing female oocytes [134]. Their involvement in pituitary POMC transcriptional regulation, if any, has not been studied. 

## 4. Summary

POMC is a life-essential gene and gives rise to several important peptides, including ACTH and the endogenous opioid β-lipotropin in the pituitary corticotroph cells. This review summarizes the roles of various NRs as well as linage-specific factors that regulate pituitary specific POMC transcription under normal and disease conditions. These studies employed the in vitro cell model, in vivo animal model as well as human subject trials and provided informative mechanisms and crosstalk between various developmental and signaling pathways. Among them, GR and ligand GCs are well documented negative regulators, however, resistance to GCs is a common phenome of pituitary corticotroph tumors and further studies revealed the involvement of chaperon Hsp90 and TR4 which warrants feasible targeted therapy [246]. In addition, RARs, LXRs, and Nur factors are well documented positive regulators POMC and exploration of pituitary specific antagonists or modulators of these factors will allow development of novel treatments for disorders of POMC transcription. In summary, the growing body of knowledge about the POMC transcriptional regulation provides the rationale for new medical treatments for POMC-associated diseases. 

## Figures and Tables

**Table 1:** Nuclear receptor superfamily proteins and their roles in POMC regulation.

Family	Gene Name	Gene Symbol	Abbreviation	Ligand	Effect on POMC Transcription	POMC Promoter Binding Site
0B	Dosage-sensitive sex reversal-adrenal hypoplasia congenital critical region on the Xchromosome, Gene 1	NR0B1	DAX1			
	Short heterodimeric partner	NR0B2	SHP			
1A	Thyroid hormone receptor-α	NR1A1	TRα	T3, T4		
	Thyroid hormone receptor-β	NR1A2	TRβ	T3, T4		
1B	Retinoic acid receptor-α	NR1B1	RARα	Retinoic Acids	RAs↓ [41,42,43,44,45,46,47] /Am80↑ [53]	
	Retinoic acid receptor-β	NR1B2	RARβ	Retinoic Acids		
	Retinoic acid receptor-γ	NR1B3	RARγ	Retinoic Acids		
1C	Peroxisome proliferator-activated receptor-α	NR1C1	PPARα	Fatty Acids		
	Peroxisome proliferator-activated receptor-β	NR1C2	PPARβ	Fatty Acids		
	Peroxisome proliferator-activated receptor-γ	NR1C3	PPARγ	Fatty Acids	Rosiglitazone↓ [67,68,69,70,71]	
1D	Reverse-Erb-α	NR1D1	REV-ERBα	Heme		
	Reverse-Erb-β	NR1D2	REV-ERBβ	Heme		
1F	Retinoic acid receptor-related orphan receptor-α	NR1F1	RORα	Cholesterol		
	Retinoic acid receptor-related orphan receptor-β	NR1F2	RORβ	Cholesterol		
	Retinoic acid receptor-related orphan receptor-γ	NR1F3	RORγ	Cholesterol		
1H	Liver X receptor-α	NR1H3	LXRα	Oxysterols	TO901317↑ [119,121]	(-73 AGGAAGGTCA CGTC CAAGGCTCA -52) [119]
	Liver X receptor-β	NR1H2	LXRβ	Oxysterols		
	Farnesoid X receptor-α	NR1H4	FXRα	Bile acids		
	Farnesoid X receptor-β	NR1H5P	FXRβ	/Farnesoids		
1I	Vitamin D receptor	VDR	VDR	Vitamin D		
	Pregnane X receptor	NR1I2	PXR	Endobiotics		
	Constitutive androstane receptor	NR1I3	CAR	/Xenobiotics		
2A	Hepatocyte nuclear factor-4-α	HNF4A	HNF4α	Fatty Acids		
	Hepatocyte nuclear factor-4-γ	HNF4G	HNF4γ	Fatty Acids		
2B	Retinoid X receptor-α	RXRA	RXRα	9cisRA	HX630 ↓ [55]	
	Retinoid X receptor-β	RXRB	RXRβ	9cisRA		
	Retinoid X receptor-γ	RXRG	RXRγ	9cisRA		
2C	Testicular orphan nuclear receptor 2	NR2C1	TR2			
	Testicular orphan nuclear receptor 4	NR2C2	TR4		MEK-162 ↓ [148]	
2E	Tailless homolog orphan receptor	NR2E1	TLX			
	Photoreceptor-cell-specific nuclear receptor	NR2E3	PNR			
2F	Chicken ovalbumin upstream promoter-transcription factor-α	NR2F1	COUP-TFα			
	Chicken ovalbumin upstream promoter-transcription factor-β	NR2F2	COUP-TFβ			
	Chicken ovalbumin upstream promoter-transcription factor-γ	NR2F6	COUP-TFγ			
3A	Estrogen receptor-α	ESR1	ERα	Estrogen	hypothalamic POMC enhancer ↑ [152]	
	Estrogen receptor-β	ESR2	ERβ	Estrogen		
3B	Estrogen-related receptor-α	ESRRA	ERRα			
	Estrogen-related receptor-β	ESRRB	ERRβ			
	Estrogen-related receptor-γ	ESRRG	ERRγ			
3C	Glucocorticoid receptor	NR3C1	GR	GC	GC ↓ [167,168,169,170]	(-63 CGTCCA -58) [167,168,169,170]
	Mineralocorticoid receptor	NR3C2	MR	GC/MC		
	Progesterone receptor	PGR	PR	Progesterone		
	Androgen receptor	AR	AR	Androgen		
4A	Nerve growth factor 1B	NR4A1	Nur77	UFAs	CRH, IL-1 ↑ [197,198,199,200,201]	(-71 GAAGGTCA -63) [197]
	Nurr-related factor 1	NR4A2	Nurr1	UFAs		(-405 TGATATTT ACCTCC AAATGCCA -384) [198]
	Neuron-derived orphan receptor-1	NR4A3	NOR-1	6-MP		
5A	Steroidogenic factor-1	NR5A1	SF1	Phospholipids		
	Liver receptor homolog-1	NR5A2	LRH-1	Phospholipids		
6A	Germ cell nuclear factor	NR6A1	GCNF			
